# Genome-resolved analyses of oligotrophic groundwater microbial communities along phenol pollution in a continuous-flow biodegradation model system

**DOI:** 10.3389/fmicb.2023.1147162

**Published:** 2023-03-29

**Authors:** Maryam Yavari-Bafghi, Maryam Rezaei Somee, Mohammad Ali Amoozegar, Seyed Mohammad Mehdi Dastgheib, Mahmoud Shavandi

**Affiliations:** ^1^Extremophiles Laboratory, Department of Microbiology, School of Biology, College of Science, University of Tehran, Tehran, Iran; ^2^Centre for Ecology and Evolution in Microbial Model Systems (EEMiS), Linnaeus University, Kalmar, Sweden; ^3^Microbiology and Biotechnology Group, Environment and Biotechnology Research Division, Research Institute of Petroleum Industry, Tehran, Iran

**Keywords:** oligotrophic groundwater, biodiversity, metagenome, phenol, bioremediation

## Abstract

Groundwater pollution is one of the major environmental concerns. The entrance of pollutants into the oligotrophic groundwater ecosystems alters native microbial community structure and metabolism. This study investigated the application of innovative Small Bioreactor Chambers and CaO_2_ nanoparticles for phenol removal within continuous-flow sand-packed columns for 6 months. Scanning electron microscopy and confocal laser scanning microscopy analysis were conducted to indicate the impact of attached biofilm on sand surfaces in bioremediation columns. Then, the influence of each method on the microbial biodiversity of the column’s groundwater was investigated by next-generation sequencing of the 16S rRNA gene. The results indicated that the simultaneous application of biostimulation and bioaugmentation completely eliminated phenol during the first 42 days. However, 80.2% of phenol remained in the natural bioremediation column at the end of the experiment. Microbial diversity was decreased by CaO_2_ injection while order-level groups known for phenol degradation such as *Rhodobacterales* and *Xanthomonadales* dominated in biostimulation columns. Genome-resolved comparative analyses of oligotrophic groundwater prokaryotic communities revealed that *Burkholderiales*, *Micrococcales*, and *Cytophagales* were the dominant members of the pristine groundwater. Six-month exposure of groundwater to phenol shifted the microbial population towards increasing the heterotrophic members of *Desulfobacterales*, *Pseudomonadales*, and *Xanthomonadales* with the degradation potential of phenol and other hydrocarbons.

## 1. Introduction

In recent decades, increasing demand for petroleum hydrocarbon, leakage from storage tanks and pipelines, and unsafe waste transportation have become severe global environmental issues. The pollutants are easily transported by the natural water flow and contaminate the downstream groundwater (Koshlaf et al., 2016; Wang et al., 2016; Lu et al., 2017). Phenols are the major organic compounds present in the effluents of various industries (e.g., petroleum), which contaminate groundwater by infiltration through the soil of the polluted area (Mohammadi et al., 2015; Sun et al., 2015). They are considered the main pollutants having high solubility in water (84.2 g L^–1^) and hence carcinogenic impacts on humans, animals, and plants (Villegas et al., 2016).

Groundwater, as a most critical water resource, is widely used in washing and irrigation processes for about 40% of the world’s agricultural products and also influences the rates and types of biogeochemical cycles in water networks (Lall et al., 2020). Although all domains of life are present and active in groundwater, archaea and bacteria showed a clear dominance over eukarya (Lopez-Fernandez et al., 2018). Prokaryotes, with estimated total abundances of 10^2^–10^6^ cells, have a vital role in the biogeochemical cycles (Clark et al., 2018). The entrance of hydrocarbon pollutants into the oligotrophic groundwater ecosystems will increase microbial metabolism, change their community structure, and affect their role in cycling elements (Flynn et al., 2008). Therefore, pollutant removal from groundwater has great importance.

Among the numerous physical, chemical, and biological groundwater treatment approaches, bioremediation has been widely used as a safe, economical, and eco-friendly method in recent years (Dong et al., 2019; Cecconet et al., 2020). However, it is a time-consuming process due to the limited amount of dissolved oxygen (DO) and low microbial count of the groundwater (Lu et al., 2017). To overcome this problem, biostimulation [by injection of oxygen-releasing compounds (ORCs)] and bioaugmentation (using exogenous microbial consortia inoculation) were applied in several studies (Pickup et al., 2001; Mikkonen et al., 2018).

Oxygen-releasing compounds, like calcium peroxide (CaO_2_), decompose to oxygen and hydroxyl radicals after exposure to water and accelerate the contaminant degradation (Mosmeri et al., 2018, 2019). According to previous studies, the generated O_2_ stimulates the intrinsic groundwater microorganisms, and OH^●^ radicals destroy the aromatic structure of pollutants (Yeh et al., 2010; Gholami et al., 2019). CaO_2_ nanoparticles have been applied for groundwater treatment in a lot of research (Cassidy and Irvine, 1999; Xue et al., 2018; Mosmeri et al., 2019). For instance, in Qian et al. (2016) study, 2,4-dichlorophenol was removed by nanoscale CaO_2_ from groundwater. On the other side, the application of bioaugmentation to treat groundwater samples polluted with phenol, arsenic, and total petroleum-hydrocarbons (TPH) has recently received increasing interest (Dey et al., 2016; Hedbavna et al., 2016; Poi et al., 2018; Zhuang and Fang, 2020). However, achieving and maintaining a sufficient microbial mass over time is the main challenge for implementing bioaugmentation. In our previous study, Small Bioreactor Chambers (SBCs) were developed that enabled high concentrations of external specific microbial consortiums to stay active (alive) and grow normally inside the 3D chambers in a suspended state for phenol removal of groundwater. The innovative design of SBCs allowed diffusion of phenol, DO, and nutrients into the encapsulated culture, however, confined the inner bacteria due to the presence of cellulose acetate membranes with pores of up to 0.22 μm in diameter. In addition, the SBCs contained a lyophilized specific consortium which was in a dry state and not active. The penetration of water through the membrane activated the freeze-dried culture and as long as the nutrients and DO were present in the water, the consortium was active and stable (Yavari-Bafghi et al., 2022). Due to the specific SBCs structure, indigenous or consortium bacteria, cannot pass through the 0.22 microfiltration (MF) membrane and as a result, SBCs are more environmentally friendly than polymer beads (González, 2001; Duan et al., 2016).

Despite the vast number of publications on the ecological characteristics of groundwater, the microbial community dynamics and the metabolic context in response to pollutant exposure during the bioremediation process, especially in Iran, have been less noticed.

Current work aims to (i) investigate the performance and impact of simultaneous use of CaO_2_ nanoparticles and SBCs on the intrinsic microorganisms of oligotrophic groundwater within phenol bioremediation during continuous-flow experiments (pilot level), (ii) survey the microbial community dynamics of the groundwater along the phenol pollution, and (iii) study the microbial potential metabolic capabilities in hydrocarbon degradation and biogeochemical cycles. To achieve these objectives, we conducted a 6-month biostimulation and bioaugmentation investigation through the sand-packed columns with a continuous flow of phenol-polluted groundwater. The genome-resolved comparative analyses of the pristine and phenol-contaminated groundwater were performed afterward.

## 2. Materials and methods

### 2.1. Chemical

Phenol (99.99%), calcium chloride (99.99%), sodium hydroxide (99.99%), sodium azide, and glutaraldehyde (25% in H_2_O, grade l) were prepared from Sigma-Aldrich, R_2_A agar, and H_2_O_2_ (30%) were purchased from Merck. All other chemicals were of analytical grade and obtained from commercial sources. The sand and groundwater used in this study were prepared as described in our previous work (Yavari-Bafghi et al., 2022).

### 2.2. Calcium peroxide nanoparticles synthesis

Calcium peroxide nanoparticles were synthesized based on calcium chloride (CaCl_2_) and hydrogen peroxide reaction conforming to the procedure described by Khodaveisi et al. (2011).

### 2.3. Column experiments

According to the results of batch experiments in our previous work (Yavari-Bafghi et al., 2022), column experiments were conducted to investigate the CaO_2_ nanoparticles and SBCs performance in phenol removal from oligotrophic groundwater through the permeable reactive barrier (PRB). Four continuous up-flow Plexiglas reactors (100 cm length and 9 cm inner diameter) were packed with underground originated acid-washed and autoclaved sands with particle size in the 2–4 mm range. The phenol (100 mg/L) contaminated groundwater was passed through the columns with a 20 cm/day flow rate. To simulate the underground conditions, all the experiments were carried out in a cold room with a temperature of 15 ± 0.5°C.

**FIGURE 1:**
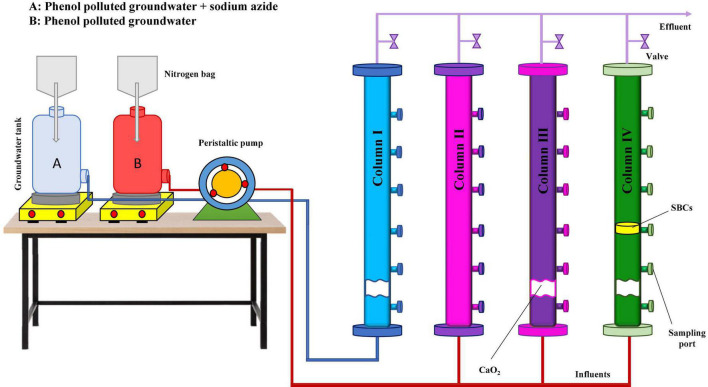
Schematic representation of column experiments. Column I: phenol removal in the abiotic condition by CaO_2_ nanoparticles (chemical), column II: natural bioremediation of phenol (blank), column III: phenol remediation by CaO_2_ nanoparticles and native groundwater microbial population (biostimulation), and column IV: phenol remediation by CaO_2_ nanoparticles and SBCs (biostimulation and bioaugmentation).

As illustrated in Figure 1, column I was considered the abiotic column in which the filter-sterilized phenol-contaminated groundwater containing 1 g/L sodium azide was used as the inlet flow to study the chemical phenol removal efficiency by calcium peroxide nanoparticles. Column II was used to simulate the natural bioremediation process as the blank column. Columns III (CaO_2_ injected) and (IV) (CaO_2_ injected + SBCs placed) were applied to investigate the efficiency of biostimulation and bioaugmentation processes, respectively in the phenol removal of groundwater.

Before the experiments, groundwater from the feed tank (pH 7.4 ± 0.01 and DO of 4 mg/L) flowed through the packed columns for 4 weeks to perform the adaptation phase. Initially, 15 g of CaO_2_ nanoparticles were injected into columns I, III, and IV. Then, to maintain the desired conditions in the columns, 15 and 5 g of CaO_2_ were re-injected after 8 and 16 weeks, respectively. SBCs were placed in column IV simultaneously with the first CaO_2_ injection. DO, pH, phenol concentration, and microbial count were measured within 6 months.

#### 2.3.1. Sampling and chemical analysis

The DO and pH of the water samples of each column effluent were measured using HQ40d multimeter (HACH). Phenol concentration in groundwater was analyzed by high-performance liquid chromatography (HPLC) (SPD-M10 A, Shimadzu, Japan). The HPLC analysis conditions were as previously presented by Yavari-Bafghi et al. (2022). Moreover, the total culturable microbial count of the effluent samples was measured by the standard plate count (SPC) method on R2A agar, and the colonies were counted by a standard colony counter (SC6 Plus, Stuart). At the end of the experiments, the physicochemical parameters of the effluent of each column were analyzed through standard methods (Supplementary Table 1) and compared with groundwater parameters reported in the authors’ previous study (Yavari-Bafghi et al., 2022).

Additionally, scanning electron microscopy (SEM) (VEGA\\TESCAN-XMU) and confocal laser scanning microscopy (CLSM) (Leica TCS SPE) were applied according to Gholami et al. (2018) and Inoué (2006), respectively, to study the microbial attachment and biofilm formation on the sand surfaces.

#### 2.3.2. DNA extraction and microbial diversity analysis

To study the effects of CaO_2_ and SBCs application on the structure and diversity of the oligotrophic groundwater microbial community by 16S rRNA gene amplicon sequencing, total microbial DNA was extracted from column’s sand surfaces using DNeasy^®^ PowerSoil^®^ (QIAGEN) according to the manufacturer’s protocol. In addition, the genomic DNA of the SBC’s consortium was extracted through the NucleoSpin^®^ Microbial DNA isolation kit (Macherey-Nagel) according to the manufacturer’s protocol to investigate the consortium dynamics during the experiment in comparison to our previous study (Yavari-Bafghi et al., 2022). The quality and concentration of the extracted DNA were analyzed by NanoDrop UV–Vis spectrophotometer (ND-1000, USA). Additionally, to ensure the structural integrity of the extracted DNA, 5 μl DNA was loaded on 1.0% agarose gel and visualized under UV in a gel documentation system. The V3–V4 region of the 16S rRNA gene was analyzed using 341F and 806R universal primers and sequenced on the Illumina MiSeq platform (Novogene company, Hong Kong).

Paired-end reads were merged to tags based on overlaps using FLASH (V1.2.7) (Magoc and Salzberg, 2011). To obtain high-quality clean tags, quality filtering on the raw tags was performed according to the Qiime (V1.7.0) (Caporaso et al., 2010). Operational taxonomic units (OTUs) were generated using Uparse software (V7.0.1090) with a 97% similarity threshold for the tags (Edgar, 2013). SSUrRNA database (SILVA138.1) was applied for species annotation of each representative sequence at each taxonomic rank, using Mothur software. The phylogenetic relationship of all sequences was obtained by MUSCLE (V3.8.31) which can compare multiple sequences rapidly (Edgar, 2004; Quast et al., 2013). Histograms of the relative abundance (%) of taxa in different samples were plotted in RStudio (V3.1.1).

### 2.4. Comparative metagenomic analyses of groundwater prokaryotic communities

#### 2.4.1. Sampling site description, sample collection, and DNA extraction

Comparative metagenomic analyses were performed during 6 months of continuous phenol pollution (100 mg/L) to investigate the dynamics of oligotrophic groundwater microbial communities due to phenol contamination. Sampling from the deep groundwater well located in the research institute of the petroleum industry, Tehran, Iran (35°44′18.7″ N 51°15′34.0″ E) was done in May 2020 using a water pump. This water sample was considered a control point without any pollution (GW). The sands used in the second column in the PRB studies were collected after 6 months of experiments. As mentioned before, the phenol (100 mg/L) contaminated groundwater was passed through the column for 6 months and the sands, which were autoclaved before applying into the column, were considered a suitable substrate for the formation of microbial biofilms during the experiment (second point, R2).

For the first sampling point, 80 L of water sample were collected and pre-filtered through 20 μm (Albet DP5891150, Germany), and 5 μm pore-size (Albet DP5895150, Germany) filters (15 cm in diameter). Microbial biomass was finally concentrated on 0.22 μm pore-size cellulose acetate filters (Sartorius 11107-142-N, Germany) using a peristaltic pump. Sand samples were collected from the sampling port of the column using sterile laboratory pincers. Water filters and sand samples were stored at −80°C until DNA extraction.

A standard phenol-chloroform protocol (Martín-Cuadrado et al., 2007) was used for extracting community DNA from the water sample. DNeasy PowerMax Soil DNA Extraction Kit (QIAGEN 12988-10, Germany) was applied for DNA extraction of the sand samples, according to the manufacturer’s instructions. Extracted DNA samples were sequenced using Illumina Novaseq 6000 platform (PE150) (Novogene, Hong Kong).

#### 2.4.2. Ribosomal RNA classification

A subset of 5 million reads was separated from each dataset, and the reads affiliated with ribosomal RNA genes were detected using SSU-ALIGN (Nawrocki, 2009). Then, the putative prokaryotic 16S rRNA gene sequences were blasted against the SILVA reference database (release 138.1 SSUParc) using BLAST, and their taxonomic affiliation was assigned based on their closest hit if the read was ≥90 bp at the similarity threshold of ≥90.

#### 2.4.3. Sequence assembly, binning, and annotation

Paired-end reads of the sequenced datasets were interleaved and quality trimmed using reformat.sh and bbduck.sh scripts of the BBMap toolkit, respectively (Bushnell, 2014). All trimmed sequences of each dataset were assembled separately using MEGAHIT (k-mer list 49, 69, 89, 109, 129, and 149) (Li et al., 2015). MetaBat2 software binned contigs ≥1 kb into metagenome-assembled genomes (MAGs) based on their different mapping depth and tetranucleotide frequency (Kang et al., 2019). The quality and completeness of the reconstructed MAGs were evaluated with CheckM (Parks et al., 2015). The taxonomy of MAGs with ≥40% completeness and ≤5% contamination was assigned using GTDB-tk (release 202) (Chaumeil et al., 2020). Putative genes were predicted with Prodigal (Hyatt et al., 2010) and preliminarily annotated using Prokka in the metagenomics mood (Seemann, 2014). Finally, the eggNOG-mapper was used to annotate further each MAG’s predicted protein sequences (Huerta-Cepas et al., 2019).

## 3. Results and discussion

### 3.1. Column experiments

#### 3.1.1. Phenol removal experiments

Four continuous-flow columns were set up to investigate the phenol removal rate from oligotrophic groundwater by CaO_2_ nanoparticles and SBCs. The experimental columns were as follows: (I) abiotic control column (CaO_2_ + sodium azide), (II) natural remediation column (without CaO_2_ and SBCs), (III) biostimulation column (CaO_2_), and (IV) simultaneous biostimulation and bioaugmentation column (CaO_2_ + SBCs).

**FIGURE 2:**
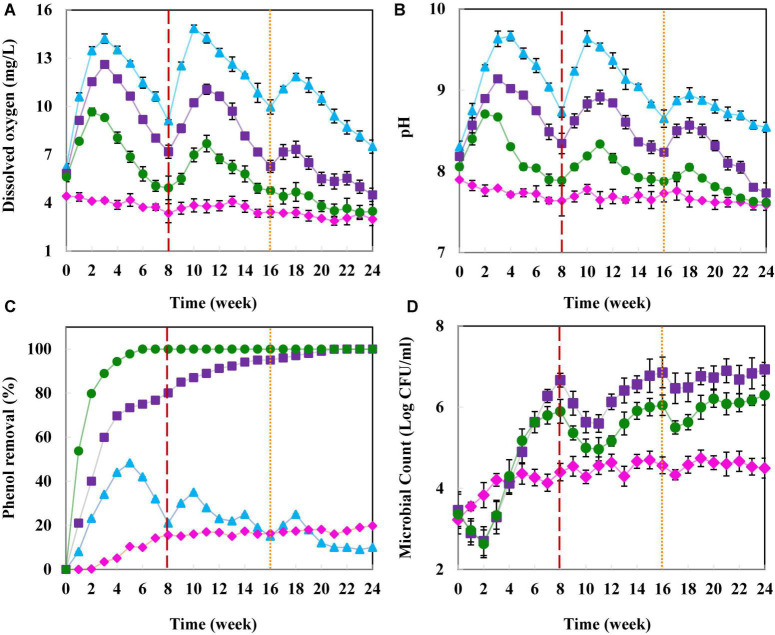
Remediation of phenol from groundwater in the column experiments. **(A)** Dissolved oxygen concentration (mg/L), **(B)** pH, **(C)** phenol removal (%), and **(D)** intrinsic microbial count (log CFU/mL). Abiotic column (▲), natural remediation column II (◆), biostimulation column III (■), and simultaneous biostimulation and bioaugmentation column IV (●). The red and orange dashed lines, respectively indicate 15 and 5 g CaO_2_ nanoparticles re-injection to provide the proper contaminant removal condition.

As presented in Figure 2, the DO in the chemical column I effluent increased notably after the first injection and peaked on the 21st day, reaching 14.14 mg/L. Production of Ca(OH)_2_ from calcium peroxide decomposition increased the pH in the injection zone (Figure 2B), resulting in the higher stability of solid peroxide (Gholami et al., 2018). According to Northup and Cassidy (2008), CaO_2_ in an alkaline environment tends to produce O_2_ rather than OH^●^. Therefore, most CaO_2_ nanoparticles in column I were converted to oxygen, which completely remained in the abiotic column.

By injection of nanoparticles into columns III and IV, the DO increased in comparison to the blank column II (Figure 2A). Within 21 days, it reached 12.62 and 9.31 mg/L in columns III and IV, respectively. However, due to the high microbial count (intrinsic and SBCs’ communities) (Figure 2D) and oxygen consumption in column IV, DO dropped sharply (Figure 2A). To overcome this problem, the oxidative agents were re-injected into the groundwater after 8 and 16 weeks of the experiment.

After the first nanoparticle injection, the pH of column I effluent significantly increased and reached to 9.17 within 4 weeks (Figure 2B). In the three other columns, the pH slowly decreased, consistent with previous studies (Gholami et al., 2018; Mosmeri et al., 2019). According to Padhi and Gokhale (2017), it seems possible that these results are due to the production of acidic metabolites from the degradation of contaminants by microorganisms.

Only 19.8% of the phenol was naturally removed from groundwater in column II within 6 months (Figure 2C). Meanwhile, the addition of CaO_2_ and SBCs to column IV resulted in the complete phenol remediation after 42 days. The contaminant removal efficiency in column III containing CaO_2_ increased to 90% after 12 weeks and reached 100% in the 22nd week. In the abiotic column I, 48.3% of the phenol was removed within 5 weeks; however, a remarkable reduction in the contaminant removal efficiency was observed afterward.

Further CaO_2_ injection into column I at weeks 8 and 16 could not improve the phenol removal by more than 48.3%. The majority of H_2_O_2_ generated from CaO_2_ was converted to oxygen in the abiotic column I, and OH^●^ content in water was not enough to degrade 100 mg/L of phenol (Figure 2C). These results were consistent with previous studies (Northup and Cassidy, 2008; Mosmeri et al., 2017; Gholami et al., 2018). Moreover, in accordance to prior surveys, our results indicated that by increasing the groundwater DO, microbial growth and activity gradually increased (Mosmeri et al., 2017; Gholami et al., 2019; Yavari-Bafghi et al., 2022).

In the natural bioremediation column I, the culturable microbial count was increased logarithmically to more than 3.2 × 10^4^ CFU/ml, then significantly limited due to the lack of enough available oxygen for microorganisms. After injecting CaO_2_ nanoparticles into the biotic columns III and IV, the microbial count dropped initially due to the negative impacts of OH radicals generated from nanoparticles on the intrinsic microorganisms. Then, the count increased by adapting the microbial population to the oxidative condition (Figure 2D).

In summary, the results showed that the phenol remediation of oligotrophic groundwater was entirely successful in the presence of CaO_2_ and SBCs. Furthermore, the simultaneous application of biostimulation and bioaugmentation methods significantly increased the contaminant removal efficiency. Our results agree with Liu N. et al. (2018), which used a PRB containing CaO_2_ to remediate nitrobenzene-contaminated groundwater and observed the highest removal rate in the presence of calcium peroxide and nitrobenzene-degrading bacteria after 20 days. Additionally, a comparison of the phenol removal in the natural remediation column II and the abiotic column I indicates the crucial role of microorganisms in the remediation process. The innovative SBCs concept of macro-encapsulation allowed the possibility of selective contaminant removal in the water using the encapsulation of a specific bacterial culture. This study proved the beneficial effects of native bacterial biostimulation and bioaugmentation with external bacteria in oligotrophic environments through continuous-flow reactors. Besides, this bioremediation procedure is considered in-situ cost-effective, environmentally friendly, and performs at high efficiencies compared to conventional *ex situ* pump and treat technologies.

#### 3.1.2. Microscopic analysis of microbial biofilm on the sand surface

Apart from the planktonic groundwater microorganisms, the attached microbial population (biofilm) on the sand surfaces also plays a key role in contaminant remediation. Therefore, bacterial adhesion to the sand surface and biofilm thickness was respectively examined by SEM and CLSM.

**FIGURE 3:**
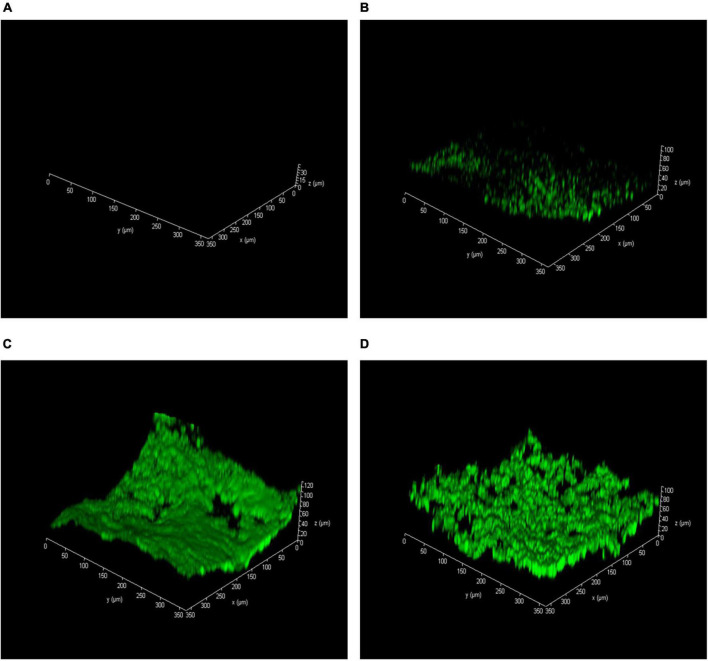
Acridine orange-stained confocal laser scanning microscopy (CLSM) images of biofilm thickness on the sand surface after 6 months. **(A)** Abiotic column I, **(B)** natural bioremediation column II, **(C)** biostimulation column III, and **(D)** biostimulation and bioaugmentation column IV.

As represented in Figure 3 and Supplementary Figure 1, no recognizable microorganism was observed on the sand surface in the abiotic column I (Figure 3A and Supplementary Figure 1A). It proved that no microbial degradation was involved in phenol removal from the column I effluent, and remediation was conducted by hydroxyl radicals (OH^●^). The groundwater microorganisms were attached to the sand surfaces and formed a thin biofilm layer (Figure 3B and Supplementary Figure 1B). However, by stimulating the microbial activity, the attached microbial communities with a thick biofilm layer can be seen on the sand surfaces (Figures 3C, D and Supplementary Figures 1C, D). It has resulted from the direct effect of oxygen-releasing nanoparticles on microbial growth. Present findings seem to be consistent with the results of Mosmeri et al. (2019) and Gholami et al. (2018), which investigated the impact of CaO_2_ on groundwater microbial communities. It can also be indicated from Figure 3C that the attached microbial communities, due to more desirable oxygen conditions in column III, formed a thicker biofilm compared to others.

#### 3.1.3. Water characteristics after treatment in each column

The concentration of NO_3_^–^, NO_2_^–^, CaCO_3_, Cl^–^, Fe (total), PO_4_^–^, color, and turbidity of the effluents from each column were examined at the end of the experiment and were compared with oligotrophic groundwater parameters (Supplementary Table 2). Among mentioned factors, PO_4_^–^, Fe (total), Cl^–^, and color showed no significant changes. However, there was a substantial decrease in the NO_3_^–^ and NO_2_^–^ concentrations of the biotic columns II, III, and IV effluent (Supplementary Table 2). These results indicate that groundwater microorganisms consume nitrate as a nitrogen source during phenol biodegradation. In line with the study of Ye et al. (2017), nitrate in groundwater can also be removed by the denitrification process.

CaCO_3_ concentration was increased from 184 mg/L in the groundwater to 253 mg/L by flowing through the abiotic column I, which contained sodium azide. However, in columns II, III, and IV effluents, the concentration of CaCO_3_ trend was decreased. According to previous reports, the coincidence of the denitrification and high pH leads to the precipitation of bicarbonates in groundwater (Dupraz et al., 2004; Hamdan et al., 2017). Meanwhile, microbial growth in columns III, IV, and II, raised the water turbidity from 1.8 to 2.5, 2.2, and 1.9, respectively. In contrast, abiotic column I effluent, which lacked microbial growth, was the most transparent sample.

#### 3.1.4. Microbial community analysis

Microbial community compositions of columns at the order level are visualized in Supplementary Figure 2. Although *Rhodobacterales* with more than 70% relative abundance were dominated in samples from columns III and IV, they were present in lower than one percent in another column. In accordance with the current results, Jehmlich et al. (2010) demonstrated that the order *Rhodobacterales* abundance increased along the chronic hydrocarbon pollution in groundwater. Therefore, the presence of this order in columns III and IV indicates its ability to remove phenol under aerobic conditions. The order *Burkholderiales* showed a high abundance (45.01%) in column II, while it had a percentage of around three in other columns. The reduction in the prevalence of order *Burkholderiales* in biostimulation columns III and IV was probably due to the toxic effects of hydroxyl radical generation, which was previously mentioned in section “3.1.1. Phenol removal experiments.”

The order *Xanthomonadales* had relatively similar frequency in all three columns. Despite the presence of *Syntrophobacterales* in the natural bioremediation column II, its frequency reached zero in the third and fourth columns. Orders *Desulfobacterales*, *Bacteriovoracales*, *Pseudomonadales*, and *Bdellovibrionales*, which were identified in column II, were not detected in columns III and IV. Representatives of *Syntrophobacterales*, *Desulfobacterales*, and *Bacteriovoracales* generally have anaerobic respiration or fermentation metabolism (Koval et al., 2015; Dyksma et al., 2018; Liu P. et al., 2018); thus, their abundance was higher in the oxygen-deficient column II than in the biostimulation columns (Supplementary Figure 2).

According to Supplementary Figure 2, column II (natural bioremediation) exhibited the highest species richness among the samples collected at the end of the experiment. It seems that the application of ORCs had a remarkable impact on the microbial diversity of two other columns (III and IV). The same results were obtained in the bioremediation of benzene-contaminated groundwater by ORC (Mosmeri et al., 2019). Although the composition of the microbial community was changed in the presence of CaO_2_ nanoparticles, it significantly increased the remediation efficiency.

Considering the environmental concerns about using the bioaugmentation method, the microbial community dynamics of the ph100 consortium were investigated at the end of the experiment. This microbial community was compared with the initial consortium’s composition which was indicated in our previous study (Yavari-Bafghi et al., 2022). According to the results, the microbial composition of the ph100 consortium was constant during the continuous-flow experiment. Representatives of *Burkholderiales* from *Gammaproteobacteria* were the dominant order with almost the same relative abundance in both samples (Supplementary Figure 3). Based on the stability of the consortium’s composition, it can be concluded that the SBCs have no microbial leakage, and only the contaminated water penetrates through the SBCs microfiltration structure membrane. As a result, the SBCs can solve a significant part of the environmental concern of the bioaugmentation method.

### 3.2. Comparative metagenomic analyses

#### 3.2.1. Prokaryotic community composition, along the phenol pollution of groundwater

**FIGURE 4:**
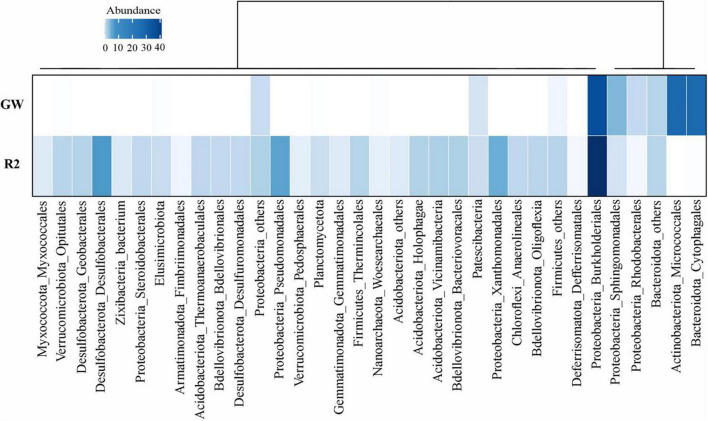
Prokaryotic community composition of the groundwater samples according to the abundance of 16S rDNA gene reads in unassembled metagenomes. Rows are the name of samples (GW: unpolluted and R2: phenol polluted groundwater). Column names are microbial taxa at the phylum and order level. For some taxa with lower frequency, the sum of orders is displayed as others or in their corresponding higher taxonomic level. There are a total number of 34 taxa for both samples. Dendrograms represent the clustering of columns based on Pearson correlation. The figure was plotted using “circlize” and “ComplexHeatmap” packages in R.

Comparative metagenomic analyses demonstrated that continuous exposure to phenol pollution in oligotrophic groundwater samples causes a shift in the microbial community composition. Hydrogen and CO_2_ are frequently found in deep groundwater ecosystems since they are formed in the Earth’s crust or mantle. Chemolithotrophs in these environments obtain their metabolic energy and carbon from the oxidation of reduced inorganic compounds and fixation of CO_2_, respectively (Purkamo, 2017). As demonstrated in Figure 4, the microbial community of pristine groundwater (GW sample) was dominated by *Burkholderiales* (*Burkholderiaceae*), *Micrococcales*, *Cytophagales*, and *Sphingomonadales*. In a previous study, it has been proved that the family *Burkholderiaceae* belongs to the order *Burkholderiales* including obligate and facultative chemolithotroph members (Pérez-Pantoja et al., 2012).

The entrance of phenol as a carbon source into the oligotrophic groundwater altered the microbial community towards a higher relative abundance of *Desulfobacterales*, *Burkholderiales* (*Rhodocyclaceae*), *Pseudomonadales*, and *Xanthomonadales* in the R2 sample.

The order *Burkholderiales* of the phylum *Proteobacteria* was the most abundant member of the community in both R2 and GW samples. Its relative abundance increased from 35.57% (*Burkholderiaceae*) in GW to 45.01% (*Rhodocyclaceae*) in R2. According to Silva et al. (2013), the most effective bacteria that harbor the metabolic pathways for phenol degradation are represented by genera from the order *Burkholderiales*.

Furthermore, sulfate-reducing bacteria (SRB) in the R2 sample comprised up to 11% of the community (*Desulfobacterales*, *Geobacterales*, *Desulfuromonadales*, and *Myxococcales*). The nitrate- and sulfate-reducing bacteria were presented in almost all samples of marine oil-polluted sediments and are considered the most crucial hydrocarbon (HC) degrading communities in aquatic ecosystems with low levels of DO (Paissé et al., 2008; Stagars et al., 2017; Rezaei Somee et al., 2021). Additionally, the orders *Pseudomonadales* and *Xanthomonadales* were prevalent in the diesel-contaminated soils and have caused the efficient removal of pollutants (Koshlaf et al., 2016). It indicated that the bioremediation process successfully raised the population of the phenol-degrading species.

Among microbial members of groundwater samples, the representatives of *Woesearchaeales* from the phylum *Nanoarchaeota* were the only archaea observed in both metagenomic samples with 1% frequency.

In accordance with the previous study that reported the negative response of *Cytophagales* to oil pollution in marine ecosystems (Rezaei Somee et al., 2021), Figure 4 represents that phenol pollution also significantly reduced the relative abundance of orders *Micrococcales* and *Cytophagales*. However, the relative abundance of *Firmicutes*, *Chloroflexi*, *Acidobacteriota*, and *Verrucomicrobiota* consistently increased from GW to R2 in response to phenol pollution. The entrance of high concentrations of HC pollutant (phenol) into the oligotrophic groundwater environment introduces new sources of carbon and more taxonomic groups were able to consume these carbon sources. As a result, the frequency of microbial groups increased and the amount of DO showed a significant reduction. In the absence of oxygen, alternative final electron acceptors such as nitrate and sulfate increased and consequently, a higher relative abundance of nitrate- and sulfate-reducing bacteria besides phenol-degrading members became dominant in the microbial community of the R2 sample. Furthermore, the alpha diversity of samples has been calculated based on the Shannon–Wiener index in R using the “vegan” package. As could be seen in Supplementary Figure 4, the R2 sample has a higher alpha index (2.3) than the pristine groundwater sample (1.4), indicating increased microbial diversity after phenol entrance.

#### 3.2.2. Genome-resolved metabolic analysis of the groundwater prokaryotic community along the phenol pollution

A total of 47 metagenome-assembled genomes (MAGs) with completeness ≥40% and contamination ≤5% were recovered from two sequenced metagenomes, among which 46 belonged to domain bacteria and one to domain Archaea. Reconstructed bacterial MAGs were mainly affiliated with *Proteobacteria* (46.8%), *Bdellovibrionota* (10.63%), *Bacteroidota* (8.51%), and *Desulfobacterota* (6.38%), along with some representatives of other phyla.

**FIGURE 5:**
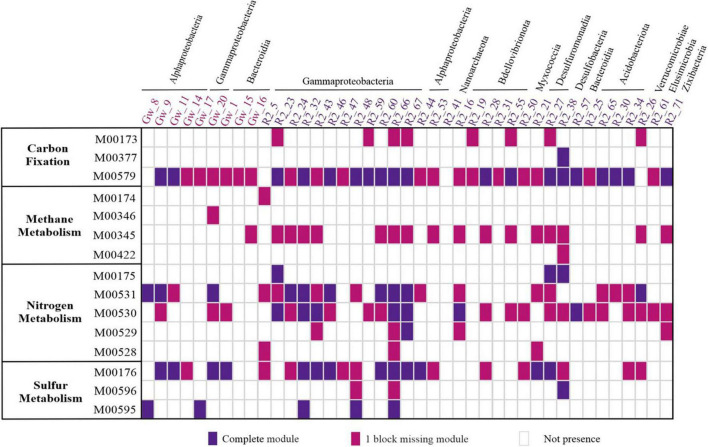
Energy metabolism pathways modules present in recovered MAGs from the groundwater metagenomes. The columns represent the taxonomy recovered of MAGs. The color of each MAG indicates the MAG origin. The row shows the cluster of metabolic pathways. Reductive citrate cycle (Arnon–Buchanan cycle) (M00173), reductive acetyl-CoA pathway (Wood–Ljungdahl pathway) (M00377), and phosphate acetyltransferase-acetate kinase pathway (M00579). Methane oxidation, methanotroph (M00174), formaldehyde assimilation, serine pathway (M00346), formaldehyde assimilation, ribulose monophosphate pathway (M00345), and Acetyl-CoA pathway (M00422). Nitrogen fixation (M00175), assimilatory nitrate reduction (M00531), dissimilatory nitrate reduction (M00530), nitrification (M00528), and denitrification (M00529). Assimilatory sulfate reduction (M00176), dissimilatory sulfate reduction (M00596), and thiosulfate oxidation by SOX complex (M00595). For the presence of a module that has all the enzymes of a pathway the value 2 and for the module that lacks one of the enzymes of a pathway the value 1 is considered.

Metabolic pathways in each MAG have been recovered based on the KEGG orthologous (KO) list of corresponding MAG via the KEGG Mapper Reconstruct website tool^1^ (Supplementary Table 3). The distribution of energy-related modules (e.g., carbon, nitrogen, and sulfur) present in at least one MAG is represented in Figure 5.

**FIGURE 6:**
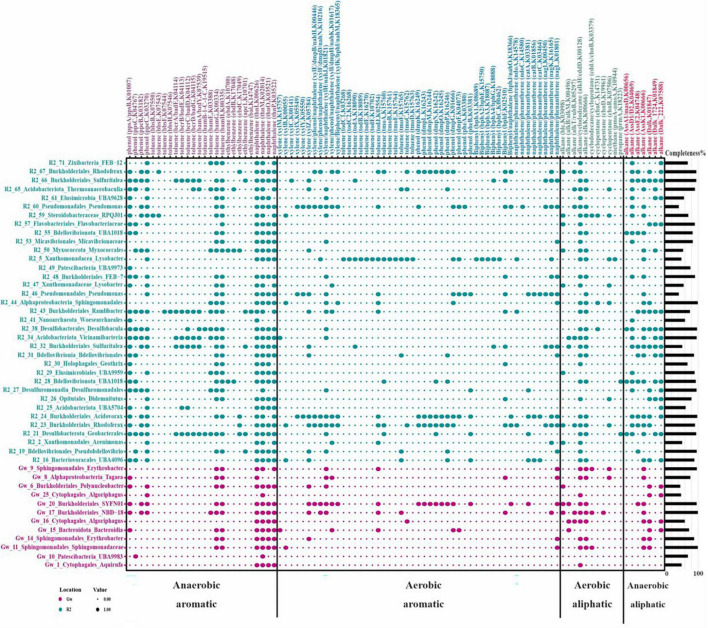
Hydrocarbon-degrading enzymes present in recovered MAGs from the groundwater metagenomes. Row names represent the taxonomy of recovered MAGs and their completeness is provided as a bar plot on the right side. The color indicates the MAG origin. Columns indicate the type of hydrocarbon and in the parenthesis is the name of the enzyme hydrolyzing this compound, followed by its corresponding KEGG orthologous accession number. The size of the dots indicates the presence or absence of each enzyme in each recovered MAG. The figure was plotted using the “reshape2” and “ggplot2” packages in RStudio.

The KEGG orthologous accession numbers (KOs) of a collection of reported enzymes involved in the degradation of different aromatic and aliphatic hydrocarbons (HCs) under aerobic and anaerobic conditions were collected and surveyed in the annotated MAGs (Abbasian et al., 2015, 2016; Rabus et al., 2016). Figure 6 represents the distribution of KEGG orthologues detected at least in one MAG (*n* = 93 genes). Mono/dioxygenases triggering the degradation of alkane, cyclododecane, biphenyl, phenol, toluene, xylene, and naphthalene/phenanthrene were detected in 43 recovered MAGs of the groundwater samples. Furthermore, the key enzymes responsible for initiating the degradation of alkane, ethylbenzene, phenol, and toluene exclusively under anaerobic conditions were detected in four reconstructed MAGs.

The metabolic context of pristine groundwater (GW sample) reconstructed MAGs suggested a primarily heterotrophic and autotrophic lifestyle. In oligotrophic groundwater, chemolithotrophs are responsible for organic matter production through CO_2_ fixation (Purkamo, 2017). Representatives of *Alphaproteobacteria* (GW_9 and GW_11) in the GW sample were autotrophs capable of fixing carbon dioxide and producing acetate by using the phosphate acetyltransferase-acetate kinase pathway (Figure 5). Acetate is a key intermediate for heterotrophic bacteria in anaerobic conditions and is also considered as a carbon source for SRBs (Dyksma et al., 2018). Furthermore, sulfur-oxidizing chemolithoautotrophic bacteria such as NBD.18 (GW_17) and *Alphaproteobacteria* member (GW_8) are probably among the primary producers in GW sample by converting thiosulfate to sulfate through sulfate oxidation with SOX complex.

According to Figure 6, the distribution of HC-degrading enzymes is less frequent in reconstructed MAGs of the GW sample. However, sulfur-oxidizing MAGs, including GW_17, GW_20 (*Burkholderiales*), as well as GW_15 and GW_16 (*Algoriphagus*), potentially have enzymes involved in the degradation of phenol, toluene, xylene, ethylbenzene, alkanes, and naphthalene under both aerobic and anaerobic conditions. They were among microorganisms with alkB/M genes and were initiating the degradation of HCs under aerobic conditions in waters (Rezaei Somee et al., 2021).

In the R2 sample, despite recovering chemolithotrophic MAGs that potentially were capable of carbon fixation (Figure 5), MAGs with HC degradation ability were more prevalent due to the high concentrations of phenol as a carbon source (Figure 6).

R2_23 and R2_24 MAGs, affiliated to *Rhodoferax* and *Acidovorax*, respectively, had the genomic potential to degrade a diverse range of HCs, including phenol, alkane, biphenyl, toluene, ethylbenzene, and xylene under both aerobic and anaerobic conditions (Figure 6). Members of *Acidovorax* have been reported to potentially degrade various organic compounds like chlorobenzene (Monferrán et al., 2005), phenanthrene (Singleton et al., 2009), trichloroethylene (Futamata et al., 2005) and polyhydroxyalkanoates (Masood, 2017). Therefore, the genus *Acidovorax* can be one of the main genera that play a crucial role in the bioremediation of a wide range of oil derivatives in polluted ecosystems.

According to previous studies, the entrance of a carbon source into groundwater causes the activation of denitrification and increases nitrate reduction (Lee et al., 2021). *Sulfuritalea* (R2_32/66), *Rhodoferax* (R2_23), *Ramlibacter* (R2_43), and *Zixibacteria* (R2_71) had denitrification potential in the R2 sample. *Sulfuritalea* members have been identified in HC-polluted environments and reported to have conserved genes of the anaerobic benzoyl-CoA pathway, such as bamA, to initiate the degradation process under anaerobic conditions (Sperfeld et al., 2018). In the present study, the existence of aromatic and aliphatic compounds degradation genes under anaerobic conditions in the *Sulfuritalea* genome was confirmed and the presence of genes related to pollutant degradation in aerobic conditions was also reported. It seems that SRB members of *Burkholderiales* and *Desulfobacteraceae*, such as *Sulfuritalea* and FEB-7, have substrate specificity in degrading hydrocarbon compounds. According to a recent study, they often degrade HCs in samples that have aliphatic compounds (Shin et al., 2019).

Representatives of *Zixibacteria* (R2_71) had genes for the degradation of phenol, toluene, ethylbenzene, alkanes, and naphthalene under anaerobic conditions. The phylum *Zixibacteria* has no cultured representative, and its genome was first introduced in 2013 by metagenomics studies of sediment samples of a reservoir near the Colorado River (USA) (Castelle et al., 2013).

Phenol, naphthalene, and phenanthrene degradation genes have also been reported for representatives of *Lysobacter* (Maeda et al., 2009; Brescia et al., 2020). According to Figure 6, the *Lysobacter* (R2_5) genome also had toluene degradation genes under aerobic conditions. However, there is no report on this ability in previous studies.

*Woesearchaeales* from *Nanoarchaeota* that have been reported in metagenomics analyses of groundwater samples (Mehrshad et al., 2021) were recovered from both GW and R2 samples and contained several enzymes contributing to phenol (ppsA/ppsB), naphthalene (thnl), and alkane (AssD1/D2) degradation under aerobic conditions.

A nitrate-reducing member of *Flavobacteriaceae* (R2_57) was recovered from the R2 sample and contained aromatic compounds’ degrading enzymes under anaerobic conditions. They mostly have enzymes that participate in the degradation of PAHs under anaerobic conditions (Rezaei Somee et al., 2021) and are potent aquatic indigenous degraders that bloom in response to HC pollution (Momper et al., 2017).

## 4. Conclusion

The present study investigated the application of SBCs and CaO2 nanoparticles in phenol-contaminated groundwater remediation through the continuous flow of sand-packed plexiglass columns. In addition, the influence of each treatment approach on the diversity and richness of the oligotrophic groundwater microbial communities was evaluated using next-generation sequencing (NGS) technology. The results indicated that applying SBCs into the simulated groundwater substrate remarkably affected the phenol removal rate. During the first 42 days of experiment, simultaneous use of CaO_2_ nanoparticles and SBCs showed great potential in the complete phenol removal efficiency. Meanwhile, the natural bioremediation process was able to remove only 19.8% of the contaminant of the column’s effluent within 6 months.

The attached microbial communities on the sand surfaces were observed by SEM and CLSM, where their crucial role in the remediation process was specified. Furthermore, studying the biodiversity of the attached biofilm in each remediation process by NGS demonstrated that the addition of CaO_2_ nanoparticles into the groundwater stimulates the contaminant biodegrading microorganisms without any adverse effect on the groundwater. Consequently, the biostimulation and bioaugmentation process is suggested for phenol-contaminated groundwater bioremediation.

Moreover, our understanding of microbial dynamics in response to phenol pollution in oligotrophic groundwater enrolled as a valuable model for advancing knowledge of managing organic and hydrocarbon spill accidents, especially in Iran. In this work, the extensive analysis of groundwater metagenome along phenol pollution illustrated the 6-month exposure to 100 mg/L phenol altered the microbial community, and microbes with the capability of pollutant degradation became the dominant population in the pollution zone. Higher-resolution analysis of the microbial community of this ecosystem in future studies can reveal critical ecological adaptations to different pollutants.

## Data availability statement

The data presented in the study are deposited in the DDBJ/EMBL/GenBank repository, accession number PRJNA741716. All data have been released and are available at https://www.ncbi.nlm.nih.gov/bioproject/?term=PRJNA741716.

## Author contributions

MS: conceptualization, supervision, review and editing, and funding acquisition. SD and MA: conceptualization, project administration, and review and editing. MY-B: methodology, investigation, software, validation, visualization, and writing—original draft. MR: software, validation, formal analysis, and review and editing. All authors contributed to the article and approved the submitted version.

## Footnotes

1
https://www.genome.jp/kegg/mapper/reconstruct.html
