# Supplementary material for: Genome-resolved analyses of oligotrophic groundwater microbial communities along phenol pollution in a continuous-flow biodegradation model system
Source: Front Microbiol. 2023 Mar 29;14:1147162. doi: 10.3389/fmicb.2023.1147162 (PMC10090433; doi:10.3389/fmicb.2023.1147162)
Supplement: Supplementary file 1 [file Data_Sheet_1.docx]

Supplementary Material

Genome-Resolved Analyses of Oligotrophic Groundwater Microbial Communities along Phenol Pollution in a Continuous-Flow Biodegradation Model System

**Maryam Yavari-Bafghi^1^, Maryam Rezaei Somee^2^, Mohammad Ali Amoozegar^1*^, Seyed Mohammad Mehdi Dastgheib^3^, Mahmoud Shavandi^3*^**

^1^Extremophiles Laboratory, Department of Microbiology, School of Biology, College of Science, University of Tehran, Tehran, Iran

^2^Centre for Ecology and Evolution in Microbial Model Systems (EEMiS), Linnaeus University, Kalmar, Sweden

^3^Microbiology and Biotechnology Group, Environment and Biotechnology Research Division, Research Institute of Petroleum Industry, Tehran, Iran

*** Correspondence:** [shavandim@ripi.ir](mailto:shavandim@ripi.ir), P. O. Box 14665-137, [amoozegar@ut.ac.ir](mailto:amoozegar@ut.ac.ir), P. O. Box 14155-6455

# Supplementary Figures and Tables

## Supplementary Figures

**Fig. S1. Scanning electron microscopic (SEM) images of biofilm formation on the sand surface after 6 months.** (a) abiotic column (I), (b) natural bioremediation column (II), (c) biostimulation column (III), and (d) biostimulation and bioaugmentation column (IV). Cocci-shaped, bacilli, and coccobacilli bacteria were determined by yellow arrows.

**Fig. S2.** **The taxonomic composition distribution histogram of column samples at the order level.** The orders with an abundance of less than 0.5% were classified into others. R2: Natural bioremediation column (II), R3: Biostimulation column (III), and R4: biostimulation and bioaugmentation column (IV).

**Fig. S3.** **The taxonomic composition distribution histogram of the ph100 consortium at a) phylum, b) class, c) order, and d) family level.** ph100 and ph100.2 represent the microbial community composition of the phenol-degrading consortium before and after use in the SBCs, respectively. The members with an abundance of less than 0.5% were named as others.

**Fig. S4.** **The alpha diversity plot of GW and R2 samples.** The R2 sample has a higher alpha index (2.3) than the pristine groundwater sample (1.4). The figure was plotted based on the Shannon-Wiener index using the “vegan” package in R.

**
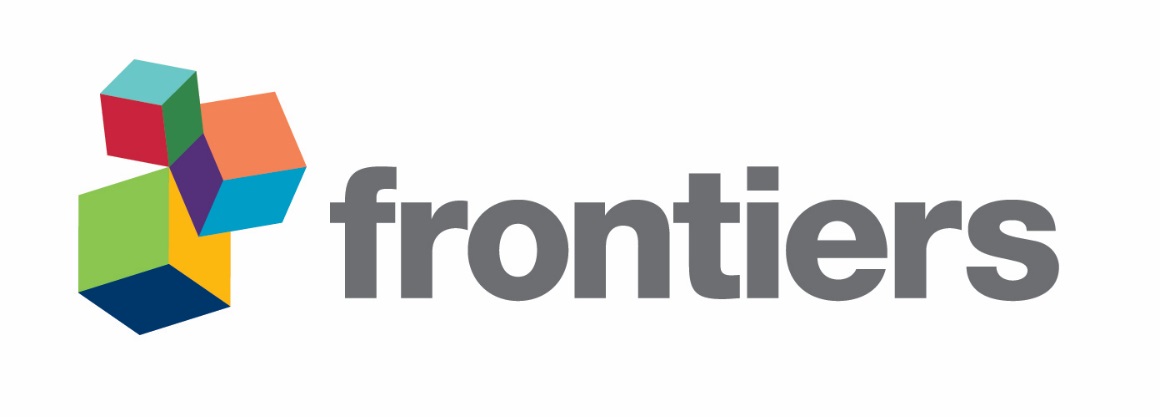
**

**Supplementary Figure S1.**

**Supplementary Figure S2.**

**Supplementary Figure S3.**

**Supplementary Figure S4.**

##
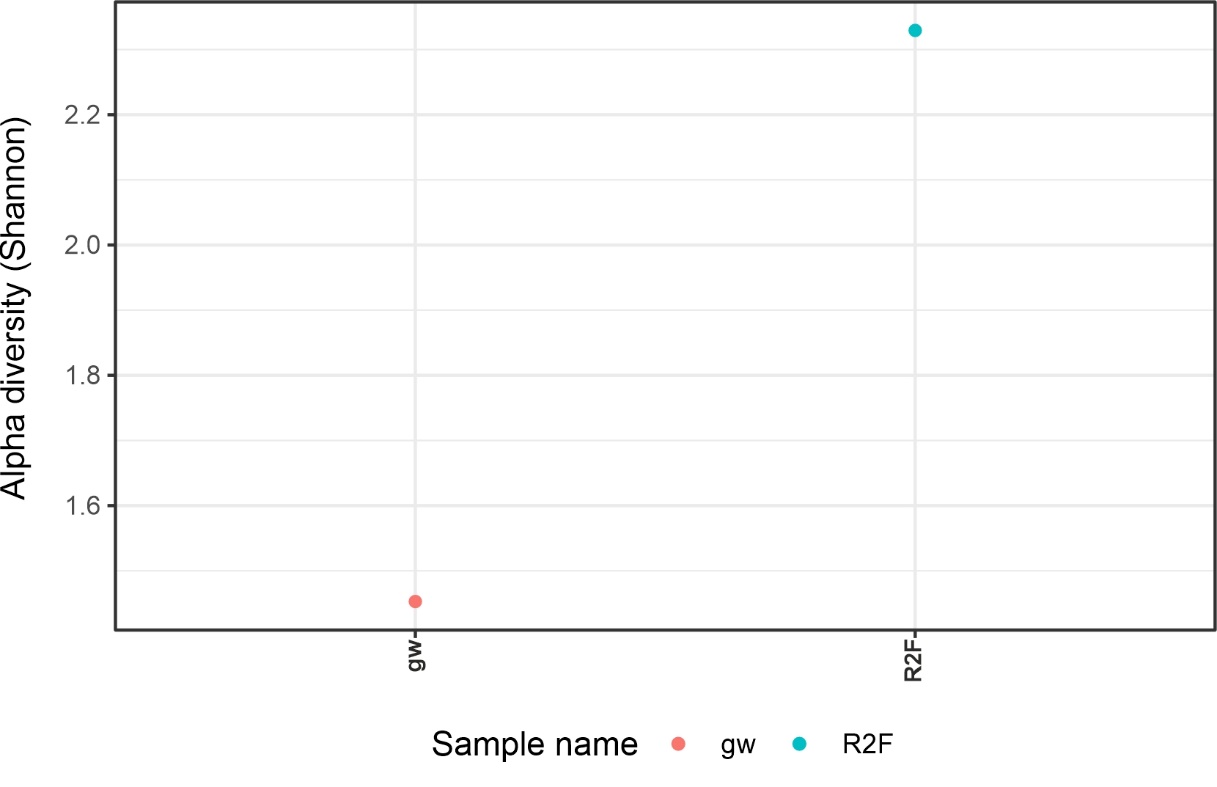


## Supplementary Tables

**Supplementary Table 1.**

**Table S1. Physicochemical analysis methods**

| **Parameter** | **Standard method** | **Parameter** | **Standard method** |
| --- | --- | --- | --- |
|  |  |  |  |
| Turbidity | ASTMD1889 | Cl^-^ | 4500-Cl-B |
| Color | 2120C | CaCO_3_ | 2340C |
| NO_3_^-^ | 4500- NO_3_-B | Fe (total) | 3500Fe |
| NO_2_^-^ | 4500- NO_2_-B | PO_4_^-^ | 4500-P |

**Supplementary Table 2.**

**Table S2. Variations in the groundwater characteristics in PRB model columns**

| **Water characteristics** | **Well water** | **Column I** | **Column II** | **Column III** | **Column IV** |
| --- | --- | --- | --- | --- | --- |
| NO_3_^-^ (mg/L) | 40 | 39.35 | < 0.1 | < 0.1 | < 0.1 |
| NO_2_^-^ (mg/L) | 0.43 | 0.42 | < 0.1 | < 0.1 | < 0.1 |
| CaCO_3_ (mg/L) | 184 | 253 | 150 | 117 | 71 |
| Cl^-^ (mg/L) | 89 | 95 | 89 | 93 | 92 |
| Fe (total) (mg/L) | < 0.05 | < 0.05 | < 0.05 | < 0.05 | < 0.05 |
| PO_4_^-^ (mg/L) | < 0.6 | < 0.6 | < 0.6 | < 0.6 | < 0.6 |
| Color (TCU) | < 5 | < 5 | < 5 | < 5 | < 5 |
| Turbidity (NTU) | 1.8 | 1.5 | 1.9 | 2.5 | 2.2 |

**Supplementary Table 3.**

**Table S3. The energy metabolism pathways modules information surveyed in the annotated MAGs of this study**

| Name | Pathway Module | Energy metabolism | NO. |
| --- | --- | --- | --- |
| Reductive citrate cycle (Arnon-Buchanan cycle) | M00173 | Carbon fixation | 1 |
| Reductive acetyl-CoA pathway (Wood-Ljungdahl pathway) | [M00377](https://www.genome.jp/module/M00377) |  |  |
| 3-Hydroxypropionate bi-cycle | M00376 |  |  |
| Hydroxypropionate-hydroxybutylate cycle | M00375 |  |  |
| Dicarboxylate-hydroxybutyrate cycle | M00374 |  |  |
| Incomplete reductive citrate cycle | M00620 |  |  |
| Phosphate acetyltransferase-acetate kinase pathway | M00579 |  |  |
| Methanogenesis | M00567 | Methane metabolism | 2 |
| Methanogenesis | M00357 |  |  |
| Methanogenesis | M00356 |  |  |
| Methanogenesis | M00563 |  |  |
| Coenzyme M biosynthesis | M00358 |  |  |
| 2-Oxocarboxylic acid chain extension | M00608 |  |  |
| Methane oxidation, methanotroph | M00174 |  |  |
| Formaldehyde assimilation, serine pathway | M00346 |  |  |
| Formaldehyde assimilation, ribulose monophosphate pathway | M00345 |  |  |
| Formaldehyde assimilation, xylulose monophosphate pathway | M00344 |  |  |
| F420 biosynthesis, archaea | M00378 |  |  |
| Methanofuran biosynthesis | M00935 |  |  |
| Acetyl-CoA pathway | M00422 |  |  |
| Nitrogen fixation | M00175 | Nitrogen metabolism | 3 |
| Assimilatory nitrate reduction | M00531 |  |  |
| Dissimilatory nitrate reduction | M00530 |  |  |
| Denitrification | M00529 |  |  |
| Nitrification | M00528 |  |  |
| Complete nitrification, comammox | M00804 |  |  |
| Assimilatory sulfate reduction | M00176 | Sulfur metabolism | 4 |
| Dissimilatory sulfate reduction | M00596 |  |  |
| Thiosulfate oxidation by SOX complex | M00595 |  |  |
